# Midterm cerebral outcomes of Stanford type A aortic dissection in patients who underwent novel triple‐branched stent graft implantation combined with intraoperative monitoring of regional cerebral oxygen saturation

**DOI:** 10.1111/jocs.14130

**Published:** 2019-07-03

**Authors:** Yong Lin, Mei‐Fang Chen, Liang‐Wan Chen, Jie‐Bo Wang, Hui Zhang, Ruo‐Meng Li

**Affiliations:** ^1^ Department of Cardiovascular Surgery Fujian Medical University Union Hospital Fuzhou Fujian Province People's Republic of China; ^2^ Department of Anesthesiology Fujian Medical University Union Hospital Fuzhou Fujian Province People's Republic of China

## Abstract

**Objective:**

The aim of this study was to evaluate the cerebral outcomes of patients underwent novel triple‐branched stent graft implantation combined with the intraoperative monitoring of regional cerebral oxygen saturation.

**Methods:**

One hundred thirty‐seven consecutive patients who underwent the surgery of triple‐branched stent graft implantation in our department were enrolled in this retrospective case‐control study. The patients in group A received brain protection based on the intraoperative monitoring of regional cerebral oxygen saturation and the patients in group B received conventional brain protection. The general clinical data, the types of corrective surgeries, the intraoperative situations, the postoperative complications, and the midterm outcomes of the patients were analyzed.

**Results:**

The incidence of postoperative cerebral dysfunction in the patients of group A was significantly lower than that in the patients in group B (3.2% vs 14.9%, *P* = .020). We found significant differences in the incubation times (30.3 ± 22.1 vs 42.3 ± 27.9 hours, *P* = .014), the lengths of intensive care unit stay (58.0 ± 54.3 vs 79.7 ± 55.5 hours, *P* = .004), and the hospital stays (19.3 ± 6.7 vs 24.9 ± 17.3 days, *P* = .045). A descending trend in the mortality rates was observed between the patients in the two groups based on the 20 months of observation; however, this trend was not statistically significant (1.6% vs 6.8%, *P* = .218).

**Conclusions:**

The novel triple‐branched stent graft implantation procedure combined with intraoperative monitoring of the regional cerebral oxygen saturation was an effective treatment for Stanford type A aortic dissection, with a relatively low incidence of postoperative cerebral dysfunction.

## 1 INTRODUCTION

Although the duration of a systemic circulatory arrest has been significantly shortened by the novel triple‐branched stent graft implantation and the cerebrovascular perfusion technique has been modified, cerebral dysfunction is still one of the most common and serious complications resulting from Stanford type A aortic dissection (AAD) surgery in our department (with rates of complications ranging from 8.6% to 10.7%)^1^, ^2^, ^3^ It is currently known that the main mechanism of postoperative cerebral dysfunction is the disbalance of oxygen metabolism in the brains of patients. Compared with conventional monitoring methods, regional cerebral oxygen saturation (rcSO_2_) can provide much earlier warning indications of cerebral hypoxia. The aim of this study was to evaluate the cerebral outcomes of patients who underwent novel triple‐branched stent graft implantation with intraoperative rcSO_2_ monitoring.

## 2 METHODS

**Figure 1:**
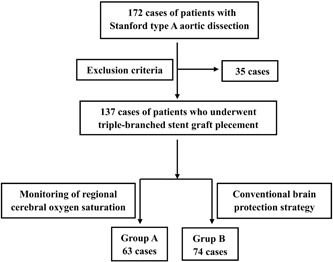
Flow Chart. The patients were divided into two groups: patients in group A received brain protection based on the intraoperative rcSO_2_ monitoring (n = 63), and patients in group B received conventional brain protection (n = 74). Thirty‐five patients were excluded from this study according to the exclusion criteria described in detail below

One hundred thirty‐seven consecutive patients with AAD who underwent triple‐branched stent graft implantation (manufactured by Yuhengjia Sci Tech Corp Ltd, Beijing, China) from January 2017 to June 2018 in our department were enrolled in this retrospective case‐control study (Figure 1). These patients were divided into two groups: the patients in group A (n = 63) received brain protection based on intraoperative rcSO_2_ monitoring, and the patients in group B (n = 74). received conventional brain protection. The general clinical data, types of corrective surgeries, intraoperative situations (eg, the value of rcSO_2_), postoperative complications (eg, anesthesia recovery period, new onset of postoperative cerebral dysfunction), and midterm outcomes of the patients were analyzed.

### 2.1 Exclusion criteria

1. Patients who were younger than 16‐years of age.
2. Patients who underwent other types of procedures (eg, Sun's procedure).
3. Patients with an abnormal preoperative consciousness.
4. Patients and/or their relatives who did not agree to participate in this clinical study.
5. Patients who were lost to the 20‐month follow‐up.

### 2.2 Diagnosis criteria of the postoperative cerebral dysfunction

Postoperative cerebral dysfunction included new‐onset stroke, syncope, delirium, postoperative cognitive dysfunction (POCD), delayed emergence from anesthesia (DEA), and coma, which were confirmed by two experienced neurologists. Deeply sedated patients, confirmed by the monitoring sedation status system of the Richmond Agitation Sedation Scale (RASS),^4^ did not receive any evaluation of cerebral function until they recovered from anesthesia.

The diagnosis of a stroke was based on the National Institutes of Health Stroke Scale.^5^ Syncope was defined as a transient loss of consciousness and was characterized by a rapid onset, short duration, and spontaneous complete recovery.^6^ The confusion assessment method for the intensive care unit^7^ was applied for the evaluation of postoperative delirium (POD) and POCD. The Glasgow Coma Scale^8^ was used to objectively define coma in the two groups of patients after their surgeries. The patients whose response to stimulation occurred more than 60 to 90 minutes after the surgeries were recognized as having a DEA,^9^ and this diagnosis should exclude the possibility of the other types of cerebral dysfunction mentioned above.

### 2.3 Protocols of anesthesia

Combined intravenous inhalation anesthesia was applied in the patients. The nasopharyngeal temperature and the rectal temperature were monitored. Transesophageal echocardiography (TEE) was applied to monitor the intraoperative hemodynamics. Autologous blood transfusion was used to reduce the allogeneic blood transfusion. The balances of cerebral oxygen metabolism of the patients were measured by rcSO_2_ with the Regional Oximetry System (INVOS 5100C, Medtronic). The bispectral index (BIS), which was measured by the BIS Monitoring System (VISTA, Covidien), was used to measure the depth of anesthesia.

During the surgery, the values of scSO_2_ were maintained at or above 70% of the baseline threshold. Cerebral desaturation was defined as a decrease in the saturation value below the absolute value of 50% or 70% of the baseline for 15 seconds. The mean and minimum values of the rcSO_2_, as well as the area under the curve (AUC) of the rcSO_2_ values below the line of the previously mentioned cerebral desaturation values, were collected for further analysis.

### 2.4 Surgical procedure

During surgery, the arterial pressures of the upper and lower limbs were monitored. A sternal incision was performed. To establish CPB, the arterial cannula was placed in the right axillary artery and the right femoral artery, and the drainage tube was placed in the right atrium. The CPB flow rate was 2.4~2.6 L·kg^−1^·min^−1^. Intermittent cold‐blood cardioplegia was perfused through the left and right coronary arteries for myocardial protection. The following procedures, including the triple‐branched stent graft implantation, can be found in our previous literature.^2^

### 2.5 Protocol to deal with intraoperative cerebral desaturation

In group A, the positions of the patients were checked first to exclude the compression of the cervical great vessels when intraoperative cerebral desaturation occurred. Then, the parameters of the mechanical ventilator (before or after CPB) or the oxygenator (during CPB) were adjusted to maintain the arterial pressure of CO_2_ (PaCO_2_) above 40 mm Hg. Metaraminol or noradrenaline was used to elevate the mean arterial pressure to above 60 mm Hg. A cardiotonic (eg, epinephrine) was administered if there was a poor cardiac index (below 2.0 L·m^−2^·min^−1^), and adequate blood volume was confirmed by TEE. The BIS value was maintained at or below 50 during the CPB to ensure that patients were in a state of deep anesthesia. Other methods to prevent cerebral hypoxia included increasing the pump flow, increasing the FiO_2_ and performing allogeneic blood transfusion. The patients in group B received conventional brain protection strategy without the guidance of intraoperative rcSO_2_ monitoring.^1^, ^2^, ^3^

### 2.6 Follow‐up

Telephone contact with the patients was maintained after discharge. Every month in the first year, the patients received echocardiographies, chest radiographies, and bilateral carotid artery Doppler examinations. At one and 3 months after surgery, the patients received aortic computed tomography angiography examinations which were then performed annually.

### 2.7 Statistical analysis

SPSS Statistics (version 19.0, IBM) was used for the statistical analyses. Descriptive statistical analyses, as well as Wilcoxon rank‐sum tests, were used to analyze the measurement data. The *χ* ^2^ test or the Fisher exact test was used to analyze the numerical data. The Kaplan‐Meier method was used to plot the survival curves. Statistical significance was defined as *P* < .05.

## 3 RESULTS

### 3.1 General clinical data

Thirty‐five patients were excluded from this study, including five cases that were younger than 16 years of age, 18 cases that underwent other types of procedures, 10 cases that were coma before surgeries, and two cases that were lost to the 20‐month follow‐up.

The primary analyses revealed that there were no significant differences between the patients in the two groups in terms of age, sex, body mass index, personal history, underlying diseases, New York Heart Association class, etiologies, ultrasound cardiogram results, preoperative complications due to aortic dissections, the scales of aortic dissections, the American Society of Anesthesiologists class and EuroSCORE II values (Table 1).

**Table 1:** Clinical data

Category of clinical data	Group A (n = 63)	Group B (n = 74)	*P* value
Age	50.6 ± 14.7	50.1 ± 11.6	.612
Sex, n (%)			.174
Male	51 (81.0)	66 (89.2)	
Female	12 (19.0)	8 (10.8)	
BMI	24.9 ± 3.7	24.2 ± 3.5	.346
Active smoking, n (%)	32 (50.8)	28 (37.8)	.128
Alcoholism, n (%)	6 (9.5)	7 (9.5)	.990
Underlying diseases, n (%)			
Diabetes	0 (0.0)	2 (2.7)	.500
CAD	0 (0.0)	1 (1.4)	1.000
Cardiac reoperation	0 (0.0)	3 (4.1)	.249
Renal dysfunction	1 (1.6)	3 (4.1)	.624
History of cerebral diseases	3 (4.8)	2 (2.7)	.661
History of anemia	4 (6.3)	5 (6.8)	1.000
NYHA class, %			
I	11	19	.681
II	41	43	
III	9	9	
IV	2	3	
Etiologies, n (%)			
Hypertension	52 (82.5)	54 (73.0)	.182
Others	11 (17.5)	20 (27.0)	
UCG, n (%)			
EF, %	61.2 ± 8.7	60.3 ± 7.4	.368
Pericardial effusion,^a^ n (%)	4 (6.3)	1 (1.4)	.180
Aortic regurgitation,^b^ n (%)	8 (12.7)	10 (13.5)	.888
Preoperative complications, n (%)			
AMI^c^	2 (3.2)	4 (5.4)	.687
Lower limb ischemia	11 (17.5)	9 (12.2)	.381
Mesenteric artery infarction^d^	5 (7.9)	4 (5.4)	.732
The scale of aortic dissection, n (%)			
Ascending aorta	4 (6.3)	3 (4.1)	.208
Aortic arch	23 (36.5)	34 (45.9)	
Descending aorta	3 (4.8)	9 (12.2)	
Abdominal aorta	22 (34.9)	15 (20.3)	
Common iliac artery	11 (17.5)	13 (17.6)	
ASA status, n (%)			
I	0 (0.0)	0 (0.0)	.342
II	0 (0.0)	0 (0.0)	
III	0 (0.0)	0 (0.0)	
IV	60 (95.2)	67 (90.5)	
V	3 (4.8)	7 (9.5)	
VI	0 (0.0)	0 (0.0)	
EuroSCORE II	9.9 ± 3.6	8.9 ± 3.4	.100

*Note*: The results demonstrated that there were no significant differences between the two groups in terms of age, sex, smoking, alcoholism, underlying diseases, etiology, cardiac function, preoperative complications, or preoperative status.

Abbreviations: AMI, acute myocardial infarction; ASA, American Society of Anesthesiologists; BIS, bispectral index; BMI, body mass index; CAD, coronary artery disease; ECG, electrocardiography; EF, ejection fraction; NYHA, New York Heart Association; UCG, ultracardiography.

^a^ Serious pericardial effusion.

^b^ Serious aortic regurgitation.

^c^ Clinical manifestations, ECG, and contents of creatine kinase and troponin in the serum that were consistent with the diagnostic criteria for acute myocardial infarction.

^d^ Confirmed by superior mesenteric artery angiography.

The *χ* ^2^ test revealed that there were no significant differences in the types of surgical procedures between the patients in the two groups. The Wilcoxon rank‐sum tests also indicated that the duration of surgery, CPB, aortic cross‐clamping, moderate hypothermic circulatory arrest (MHCA) + selective cerebral perfusion (SCP), the mean value of the intraoperative BIS index, the volumes of blood loss, and perioperative allogeneic transfusion had no significant differences in the patients in these two groups (Table 2).

**Table 2:** Surgical and perioperative treatments

Categories of perioperative treatments	Group A (n = 63)	Group B (n = 74)	*P* value
Types of surgical correction, n (%)			
Aortic sinus reconstruction	7 (11.1)	14 (18.9)	.206
Bentall	5 (7.9)	8 (10.8)	.567
Wheat	1 (1.6)	1 (1.4)	1.000
Hemiarch replacement	62 (98.4)	68 (91.9)	.124
Ascending aorta replacement	61 (96.8)	68 (91.9)	.288
CABG	0 (0.0)	1 (1.4)	1.000
Intraoperative conditions			
Surgery, min	287.1 ± 64.1	285.4 ± 48.5	.743
CPB, min	139.6 ± 32.4	140.6 ± 30.3	.953
Aortic cross‐clamping, min	48.7 ± 7.7	50.0 ± 10.8	.168
MHCA + SCP, min	16.3 ± 2.2	17.0 ± 2.5	.099
Blood loss, mL	339.7 ± 144.8	408.1 ± 210.5	.155
rcSO_2_ baseline (L), %	63.2 ± 8.1	–	–
rcSO_2_ baseline (R), %	60.2 ± 9.1	–	–
Avg rcSO_2_ (L), %	64.4 ± 6.0	–	–
Avg rcSO_2_ (R), %	61.3 ± 6.9	–	–
rcSO_2_ minimum, %	45.8 ± 12.5	–	–
Total time of rcSO_2_ < 70% baseline and >15 s, n	4.4 ± 2.6	–	–
Total time of rcSO_2_ < 50% and >15 s, n	1.3 ± 1.6	–	–
AUC of rcSO_2_ < 70% (L), %min	66.2 ± 39.5	–	–
AUC of rcSO_2_ < 70% (R), %min	50.5 ± 31.3	–	–
AUC of rcSO_2_ < 50% (L), %min	27.1 ± 32.5	–	–
AUC of rcSO_2_ < 50% (R), %min	25.8 ± 24.8	–	–
Avg BIS index	40.6 ± 9.2	38.7 ± 9.3	.191
Perioperative allogeneic transfusion			
RBC, *µ*	3.3 ± 2.5	3.0 ± 3.0	.187
PLT, *µ*	1.4 ± 2.1	1.0 ± 1.3	.266
FFP, mL	285.8 ± 324.1	364.3 ± 413.7	.228
CP, U	1.4 ± 2.6	1.3 ± 2.7	.412

*Note*: The results demonstrated that there were no significant differences between the two groups in terms of types of surgical corrections, intraoperative conditions, BIS indexes, and perioperative allogeneic transfusions.

Abbreviations: AUC, area under curve; BIS, bispectral index; CABG, coronary artery bypass grafting; CP, cryoprecipitation; CPB, cardiopulmonary bypass; FFP, fresh frozen plasma; MHCA, moderate hypothermic circulatory arrest; PLT, platelet; RBC, red blood cell; rcSO_2_, regional cerebral oxygen saturation; SCP, selective cerebral perfusion.

### 3.2 Intraoperative rcSO_2_ value

**Figure 2:**
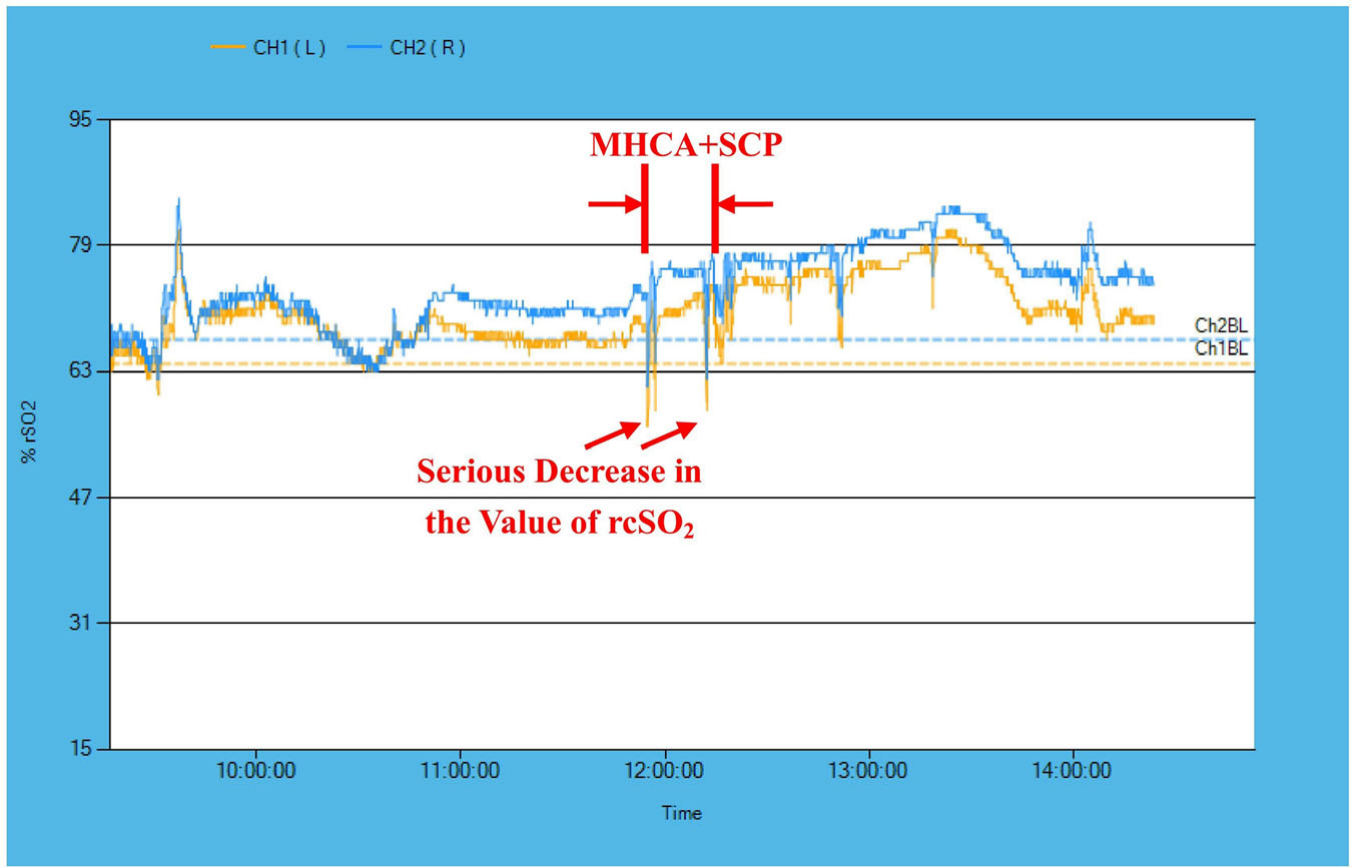
The trendgram of intraoperative rcSO_2_ value. The trendgraph of the intraoperative rcSO_2_ values displayed the variations of the intraoperative rcSO_2_ values in one classic case of a patient who underwent a triple‐branched stent graft implantation. BL, baseline; L, left brain; MHCA, moderate hypothermic circulatory arrest; R, right brain; rsSO2, regional oxygen saturation; SCP, selective cerebral perfusion

The patients in group A received intraoperative rcSO_2_ monitoring, and the baseline values of rcSO_2_ were in the normal range; the average values during the surgeries were 64.4% ± 6.0% (L) and 61.3% ± 6.9% (R), and the minimum value was 45.8% ± 12.5%. The frequency of the rcSO_2_ declined to the level of 70% of baseline (which lasted for at least 15 seconds) was 4.4 ± 2.6 times in a single operation, and the number reached a value of 1.3 ± 1.6 when the level decreased to 50% of baseline. Further analysis of the rcSO_2_ value revealed that the AUCs of the rcSO_2_ below the level of 70% of baseline were 66.2% ± 39.5% min (L) and 50.5% ± 31.3% min (R), and the AUC changed to 27.1% ± 32.5% min (L) and 25.8% ± 24.8% min (R) when the level decreased to 50% of baseline (Table 2). At the beginning of the induction, an obvious peak value of rcSO_2_ could be observed due to the inhalation of 100% oxygen before intubations. Afterward, we observed a slow decline in the rcSO_2_ value, which was associated with the establishment of the CPB. The rcSO_2_ value became relatively steady until the initiation of MHCA and SCP. The duration of the low‐flow cerebral perfusion or circulatory arrest of the brain was accompanied by a conspicuous and sharp decrease in the rcSO_2_ value (Figure 2). We immediately initiated the previously mentioned protocol to treat intraoperative cerebral desaturation. Some transient declines in the rcSO_2_ values could also be observed after the duration of MHCA and SCP, but the amplitudes were relatively mild and did not reach the cautionary line of 70% of baseline.

### 3.3 Postoperative situation

Thirteen (13 of 137, 9.5%) patients with postoperative cerebral complications were observed. The Wilcoxon rank‐sum tests showed that the anesthesia recovery periods of the patients in group A were notably shorter than those of the patients in group B, and the Fisher exact test showed that there were significant differences in postoperative cerebral dysfunction between the patients in the two groups. One case of POD and one case of POCD were discovered in the patients in group A. There were two cases of new‐onset stroke, one case of syncope, two cases of delirium, one case of POCD, two cases of DEA, and two cases of coma in the patients in group B. Two patients with postoperative paraplegia were also observed in group B, but no significant differences were found between the two groups. We also discovered significant differences in intubation time, length of stay in the intensive care unit (ICU), and length of hospital stay (Table 3).

**Table 3:** Short‐term outcomes

Category	Group A (n = 63)	Group B (n = 74)	*P* value
Anesthesia recovery period, h^a^	9.0 ± 4.3	12.8 ± 14.0	.050
New‐onset stroke, n (%)	0 (0.0)	2 (2.7)	.500
Syncope, n (%)	0 (0.0)	1 (1.4)	1.000
POD, n (%)	1 (1.6)	2 (2.7)	1.000
POCD, n (%)	1 (1.6)	1 (1.4)	1.000
DEA, n (%)	0 (0.0)	2 (2.7)	.500
Coma, n (%)	0 (0.0)	2 (2.7)	.500
Total of cerebral complications, n (%)	2 (3.2)	11 (14.9)	.020
Paraplegia, n (%)	0 (0.0)	2 (2.7)	.500
Reoperation for bleeding, n (%)	0 (0.0)	2 (2.7)	.500
Heart dysfunction, n (%)^b^	5 (7.9)	3 (4.1)	.470
Myocardial infarction, n (%)	0 (0.0)	2 (2.7)	.500
Lethal arrhythmia, n (%)	0 (0.0)	1 (1.4)	1.000
Renal insufficiency, n (%)^c^	7 (11.0)	7 (9.5)	.750
Pulmonary infection, n (%)	5 (7.9)	10 (13.5)	.297
Gastrointestinal complications, n (%)^e^	4 (6.3)	6 (8.1)	.692
Wound infection, n (%)	0 (0.0)	2 (2.7)	.500
Sepsis, n (%)	2 (3.2)	5 (6.8)	.452
ARDS, n (%)	2 (3.2)	2 (2.7)	1.000
MODS, n (%)	3 (4.8)	7 (9.5)	.284
Mechanical assistance, n (%)			
IABP	0 (0.0)	2 (2.7)	.500
ECMO	0 (0.0)	2 (2.7)	.500
Thoracic drainage^f^	469.5 ± 423.4	494.6 ± 538.9	.700
Intubation time, h	30.3 ± 22.1	42.3 ± 27.9	.014
Tracheotomy, n (%)	6 (9.5)	8 (10.8)	.804
Length of ICU stay, h	58.0 ± 54.3	79.7 ± 55.5	.004
Length of hospital stay, d	19.3 ± 6.7	24.9 ± 17.3	.045
Mortality in hospital, n (%)	1 (1.6)	3 (4.1)	.624
Mortality after discharge, n (%)	0 (0.0)	2 (2.7)	.500
Mortality after surgery, n (%)	1 (1.6)	5 (6.8)	.218
Hospital costs (RMB)	213,406.0 ± 59,481.5	238,682.3 ± 64,784.6	.019

*Note*: Patients from the rcRO_2_ group (group A) were observed to have experienced shorter anesthesia recovery periods, shorter intubation times, shorter ICU stays, shorter postoperative hospital stays, and lower rates of postoperative cerebral complications and postoperative mortalities. Patients from the rcRO_2_ group (group A) spent less money, compared with the patients in the conventional monitoring group (group B). The results demonstrated that there were no significant differences between the two groups in terms of paraplegia and non‐neurological complications after the surgeries.

Abbreviations: ARDS, acute respiratory distress syndrome; DEA, delayed emergence from anesthesia; ECMO, extracorporeal membrane oxygenation; IABP, intra‐aortic balloon pumping; ICU, intensive care unit; MODS, multiple organ dysfunction syndrome; NYHA, New York Heart Association; POCD, postoperative cognitive dysfunction; POD, postoperative delirium.

^a^ Two patients with postoperative comas were not enrolled in the analysis of recovery times.

^b^ Severe heart failure reached NYHA grades III‐IV.

^c^ Required renal replacement therapy.

^e^ Included meteorism, nausea, vomiting, abdominal pain, diarrhea, constipation, and gastrointestinal hemorrhage.

^f^ Within 48 hours after surgery.

The non‐neurological complications observed after the surgeries included reoperations for bleeding, heart dysfunction, myocardial infarction, lethal arrhythmia, renal insufficiency, pulmonary infection, gastrointestinal complications, wound infection, sepsis, acute respiratory distress syndrome, and multiple organ dysfunction syndrome. The rates of the use of intra‐aortic balloon pumping and/or extracorporeal membrane oxygenation were confirmed to be proximate between the two groups (Table 3).

Six cases of death were observed in this study, including four cases that occurred in the hospital and two cases that occurred after discharge. The Fisher exact test showed that there was no significant difference in the mortalities of the patients in the two groups, but we observed a decreasing trend in mortality after the surgeries that involved intraoperative rscO_2_ monitoring. Wilcoxon rank‐sum tests also showed that patients in group A spent markedly less money than the patients in group B (Table 3).

### 3.4 Follow‐up

One patient was lost to the 20‐month follow‐up and was excluded from this study. Two incidences of death after discharge (both of them occurring in group B) were observed and were attributed to low cardiac output syndrome and recrudescence of the aortic dissection.

### 3.5 Survival curves

**Figure 3:**
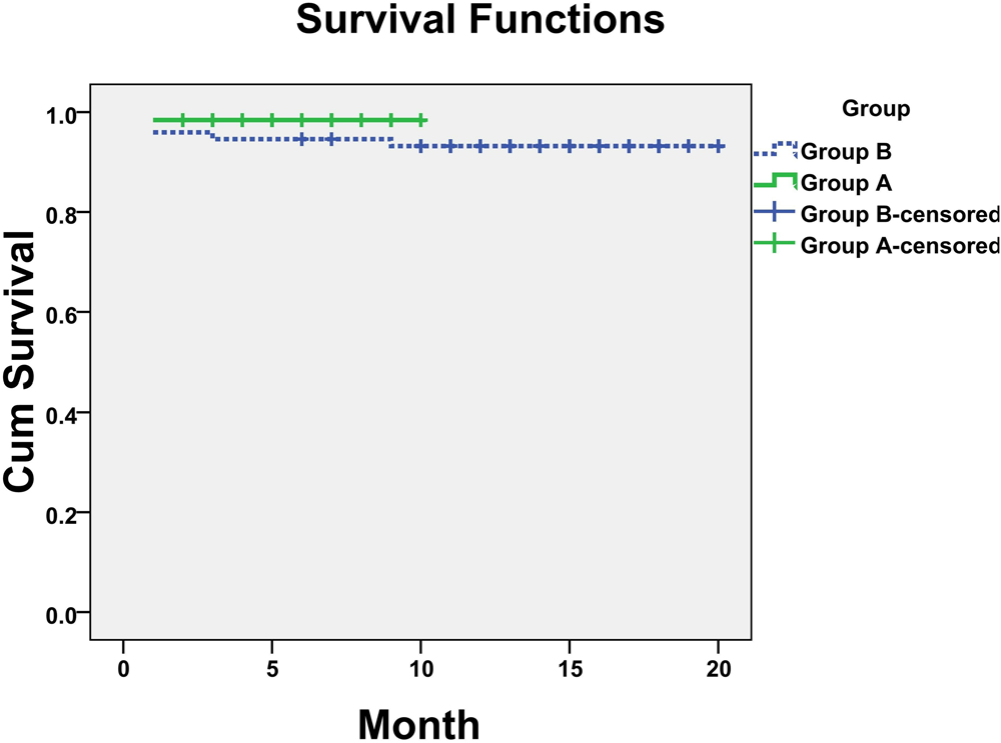
Survival Curve. Kaplan‐Meier plots revealed that a significant difference in the survival rates between patients in group A and patients in group B within the 20‐month follow‐up period. Group A, patients received rcSO_2_ monitoring; Group B, patients received conventional brain monitoring. log‐rank result: *χ* ^2^ = 1.518, *P* = .218

Survival curve analyses based on 20 months of observation revealed no significant difference in the survival rate between the patients in the two groups (Figure 3).

## 4 DISCUSSION

Postoperative cerebral dysfunction is still one of the most common complications after aortic dissection surgery, and this dysfunction can dramatically affect patient outcomes.^10^ The main mechanism of cerebral dysfunction is the disbalance of oxygen metabolism in the brains of the patients during the surgeries^11^, ^12^ Therefore, the conventional method for avoiding postoperative cerebral complications is to reduce the duration of MHCA + SCP to as short of a time as possible. Eight years ago, our department first reported a novel surgical procedure named triple‐branched stented graft implantation, which could be applied to cure AAD without a long duration of systemic circulatory arrest and without a large amount of intraoperative blood loss.^13^ However, the incidence of postoperative cerebral dysfunction is still frequent and has become a key factor that limits the increase in patients’ survival rates in our center.

In our study, 11 patients (11 of 74, 14.9%) with postoperative cerebral complications were observed in the conventional brain protection strategy group (group B). However, only two patients (2 of 63, 3.2%) were found to be suffering from brain deficits after the surgeries in group A. The most important demarcation between the patients of the two groups was the intraoperative rcSO_2_ monitoring, especially during the period of MHCA + SCP, and there were no significant differences in terms of clinical data, surgical treatments, and perioperative treatments. Although the survival rates of the patients after the surgeries between the two groups had no significant difference (1 of 63,1.6% vs 5 of 74,6.8%) based on the 20‐month follow‐up period, we still observed a descending trend in the mortality rates after the surgeries with the utilization of intraoperative rcSO_2_ monitoring. Nonetheless, we observed improved prognoses among patients who underwent corrective surgeries of AAD with the application of intraoperative rcSO_2_ monitoring. Since the intraoperative rcSO_2_ monitoring seemed to be critical in reducing the morbidity of the postoperative cerebral complications, rcSO_2_ monitoring was then performed as one of the routine intraoperative monitoring projects in aortic dissection surgeries after October 2017 in our center.

Compared with conventional monitoring methods, intraoperative rcSO_2_ monitoring can provide earlier warning indications of cerebral hypoxia and can promote immediate decision‐making in dealing with intraoperative cerebral desaturation.^14^ During the period of circulatory arrest, we observed drastic declines in rcSO_2_ (Figure 2) during the course of SCP, and the protocol was initiated immediately to treat the intraoperative cerebral desaturation. This event of brain hypoxia indicated that the utilization of hypothermia and SCP was not always reliable in protecting the brain from hypoxia, even if the duration of circulatory arrest was shortened to only 16.3 to 17.0 minutes by our novel procedure of triple‐branched stent graft implantation. There were several hypotheses to explain this phenomenon. First, a majority (73%) of the patients in China do not have an integral circle of Willis,^15^ which will result in the dysfunction of cross‐circulation at the skull base. Second, intracranial artery atherosclerosis caused by uncontrolled hypertension, hyperglycemia, hypercholesterolemia, and obesity, which frequently occurred in the patients undergoing aortic dissection, may further impair the insufficient cerebrovascular cross‐circulation^16^, ^17^, ^18^ Therefore, an SCP will not meet the oxygen demand of the different regions of the brain. Third, the hypothermia and CPB methods may depress the regulatory functions of blood flow in the brain and may induce cerebral ischemia during the SCP process. A normal level of perfusion pressure or blood flow rate may mask the malperfusion of the central nervous system.^19^ Therefore, the intraoperative rcSO_2_ monitoring has been routinely used in our center during aortic surgeries since August 2017.

In the research of short‐term outcomes, we observed shorter durations of recovery and intubation time in the patents in group A, and these results can be explained to be benefits of a relatively lower rate of morbidity due to cerebral oxygen desaturation with the application of intraoperative rcSO_2_ monitoring. Therefore, it seemed reasonable that the patients in group A experienced shorter lengths of ICU stay and shorter lengths of hospital stay. Otherwise, no significant differences were detected in terms of postoperative paraplegia or nonneural complications between the patients in the two groups.

Although the monitoring of rcSO_2_ was applied in the surgeries, two patients (1 case of delirium and 1 case of POCD) with postoperative cerebral complications were discovered in this study. In addition to the side effects of the anesthetics, intraoperative stress, electrolyte disturbance, or acid‐base imbalance, we should also consider the disadvantage of rcSO_2_ monitoring, in that this monitoring only focuses on the disbalance of oxygen metabolism in the bilateral frontal lobe, and is not sensitive to the abnormal statuses in the other regions of the brain. It would be much more consummate to make use of a whole‐brain oxygen saturation monitoring method and real‐time detection of the bilateral cerebrovascular blood flow by transcranial Doppler sonography during triple‐branched stent graft implantation surgeries.^20^ Thus, the cerebral protective effect of modified intraoperative monitoring procedures requires further validation, with larger sample sizes and more randomized controlled testing.

Furthermore, this study has several limitations. First, this retrospective case‐control and single‐center study could not ensure that the patients from the two groups received uniform therapeutic strategies. The selection bias from the subjective judgment of the physicians should not be ignored. Second, due to the small sample size, the absolute quantity of the patients with postoperative cerebral complications was relatively small and might have increased the risk of statistical errors. Third, we could not exclude the possibility of underestimated mortalities, which were attributed to the relatively short duration of the follow‐up period; therefore, a longer period of postoperative observation is required.

The novel triple‐branched stent graft implantation combined with an intraoperative cerebral protection strategy based on the rcSO_2_ monitoring could reduce the postoperative mortality and the morbidity of cerebral dysfunction. Larger sample sizes and more randomized, controlled tests are required for further validation of these procedures.

## AUTHOR CONTRIBUTIONS

Concept/design: YL and L‐WC; Data analysis/interpretation: M‐FC; Drafting article: YL; Critical revision of the article: L‐WC; Statistics: J‐BW; Data collection: HZ and R‐ML

## References

[1] Chen LW , Lu L , Dai XF , et al. Total arch repair with open triple‐branched stent graft placement for acute type A aortic dissection: experience with 122 patients. J Thorac Cardiovasc Surg. 2014;148:521‐528. 24280711 10.1016/j.jtcvs.2013.10.021

[2] Chen LW , Dai XF , Wu XJ , et al. Ascending aorta and hemiarch replacement combined with modified triple‐branched stent graft implantation for repair of acute DeBakey type I aortic dissection. Ann Thorac Surg. 2017;103:595‐601. 27553503 10.1016/j.athoracsur.2016.06.017

[3] Chen LW , Wu XJ , Dai XF , et al. A self‐adaptive triple‐branched stent graft for arch repair during open type A dissection surgery. J Thorac Cardiovasc Surg. 2015;149:1278‐1283. 25598526 10.1016/j.jtcvs.2014.11.079

[4] Ely EW , Truman B , Shintani A , et al. Monitoring sedation status over time in ICU patients: reliability and validity of the Richmond Agitation‐Sedation Scale (RASS). JAMA. 2003;289:2983‐2991. 12799407 10.1001/jama.289.22.2983

[5] Powers WJ , Rabinstein AA , Ackerson T , et al. 2018 guidelines for the early management of patients with acute ischemic stroke: a guideline for healthcare professionals from the American Heart Association/American Stroke Association. Stroke. 2018;49:e46‐e110. 29367334 10.1161/STR.0000000000000158

[6] Brignole M , Moya A , de Lange FJ , et al. 2018 ESC Guidelines for the diagnosis and management of syncope. Eur Heart J. 2018;39:1883‐1948. 29562304 10.1093/eurheartj/ehy037

[7] Ely EW , Margolin R , Francis J , et al. Evaluation of delirium in critically ill patients: validation of the Confusion Assessment Method for the Intensive Care Unit (CAM‐ICU). Crit Care Med. 2001;29:1370‐1379. 11445689 10.1097/00003246-200107000-00012

[8] Teasdale G , Jennett B . Assessment of coma and impaired consciousness. A practical scale. Lancet. 1974;2:81‐84. 4136544 10.1016/s0140-6736(74)91639-0

[9] Frost EA . Differential diagnosis of delayed awakening from general anesthesia: a review. Middle East J Anaesthesiol. 2014;22:537‐548. 25668997

[10] Bachet J . What is the best method for brain protection in surgery of the aortic arch? Selective antegrade cerebral perfusion. Cardiol Clin. 2010;28:389‐401. 20452558 10.1016/j.ccl.2010.01.014

[11] Mak NT , Iqbal S , de Varennes B , Khwaja K . Outcomes of post‐cardiac surgery patients with persistent hyperlactatemia in the intensive care unit: a matched cohort study. J Cardiothorac Surg. 2016;11:33. 26906890 10.1186/s13019-016-0411-5 PMC4765137

[12] Endlich M , Hamiko M , Gestrich C , et al. Long‐term outcome and quality of life in aortic type A dissection survivors. Thorac Cardiovasc Surg. 2016;64:91‐99. 25865779 10.1055/s-0035-1548734

[13] Chen LW , Dai XF , Lu L , Zhang GC , Cao H . Extensive primary repair of the thoracic aorta in acute type A aortic dissection by means of ascending aorta replacement combined with open placement of triple‐branched stent graft: early results. Circulation. 2010;122:1373‐1378. 20855660 10.1161/CIRCULATIONAHA.110.946012

[14] Zheng F , Sheinberg R , Yee MS , Ono M , Zheng Y , Hogue CW . Cerebral near‐infrared spectroscopy monitoring and neurologic outcomes in adult cardiac surgery patients: a systematic review. Anesth Analg. 2013;116:663‐676. 23267000 10.1213/ANE.0b013e318277a255 PMC3863709

[15] Li Q , Li J , Lv F , Li K , Luo T , Xie P . A multidetector CT angiography study of variations in the circle of Willis in a Chinese population. J Clin Neurosci. 2011;18:379‐383. 21251838 10.1016/j.jocn.2010.07.137

[16] Bornfeldt KE , Tabas I . Insulin resistance, hyperglycemia, and atherosclerosis. Cell Metab. 2011;14:575‐485. 22055501 10.1016/j.cmet.2011.07.015 PMC3217209

[17] Bos D , van der Rijk MJ , Geeraedts TE , et al. Intracranial carotid artery atherosclerosis: prevalence and risk factors in the general population. Stroke. 2012;43:1878‐1884. 22569939 10.1161/STROKEAHA.111.648667

[18] van Rooy MJ , Pretorius E . Obesity, hypertension and hypercholesterolemia as risk factors for atherosclerosis leading to ischemic events. Curr Med Chem. 2014;21:2121‐2129. 24372218 10.2174/0929867321666131227162950

[19] Klinkova AS , Kamenskaia OV , Cherniavskiĭ AM , Lomivorotov VV . Risk of development of neurological complications in prosthetic repair of the aortic ascending portion and arch. AngiolSosudKhir. 2017;23:124‐135. 28574047

[20] Wang X , Ji B , Yang B , et al. Real‐time continuous neuromonitoring combines transcranial cerebral Doppler with near‐infrared spectroscopy cerebral oxygen saturation during total aortic arch replacement procedure: a pilot study. ASAIO J. 2012;58:122‐126. 22370681 10.1097/MAT.0b013e318241abd3

